# Delayed Total Breast Reconstruction with Brava

**DOI:** 10.1155/2015/601904

**Published:** 2015-02-22

**Authors:** Niels Hammer-Hansen, Thomas Bo Jensen, Tine Engberg Damsgaard

**Affiliations:** Plastic Surgical Research Unit, Department of Plastic Surgery, Aarhus University Hospital, 33186 Aarhus, Denmark

## Abstract

Several surgical procedures exist in regard to reconstruction of the breast after mastectomy. The use of Brava a vacuum-based external soft-tissue expansion system in combination with fat transplantation is a less documented but viable option in treating patients after mastectomy. We share our experience in treating a 57-year-old patient with mastectomy, describing the complications and pitfalls we experienced when using the Brava system in combination with fat transplantation.

## 1. Introduction

Brava (Brava, LLC, Miami, FL) a vacuum-based external soft-tissue expansion system was initially developed for breast augmentation without surgical intervention [1]. The Brava system is however now used to preexpand recipient areas and thus facilitate the transplantation and survival of larger volumes of fat. Several studies have shown convincing results, the vast majority of which focus on aesthetic augmentation, congenital deformity, iatrogenic deformity, and growth related asymmetries. These treatments typically require three or fewer series of Brava in combination with fat transplantation [2–5]. The combination of external soft-tissue expansion and fat transplantation is believed to be advantageous because of the increased volume and vascularity of the recipient area created by the vacuum-based system which allows microribbons of fat to distribute more diffusely, favoring vascularization and hence survival of the fat graft [6]. In regard to complications to the Brava system few have been reported; however recently irritative skin lesions due to the use of the Brava system have been observed [7].

## 2. Case Report

A 57-year-old ethnic Danish woman was admitted to the Department of Plastic Surgery at Aarhus University Hospital for delayed reconstruction of her right breast. Two years earlier the patient had had a mastectomy of the right breast due to ductal carcinoma in situ (DCIS) and Morbus Paget of the breast. The patient had undergone adjuvant therapy with chemotherapy and Trastuzumab and received treatment with letrozole at the time of the first operation. At no time had the patient received radiation treatment of the breast. In addition to letrozole, the patient used Acrivastine and Pseudoephedrine for rhinitis, as well as Simvastatin for hypercholesterolemia and multivitamins. There was no medical history of allergic reactions or dermatological disease, although a rash had previously been noted in conjunction with the use of micropore scar tape. Upon proper information the patient opted for delayed breast reconstruction with autologous fat transfer and the Brava system.

The patient was prescribed the use of Brava for four weeks prior to autologous fat transplantation: 10 hours a day for the first 3 weeks, and 22 hours a day in the week prior to operation. It was possible for the patient to use the Brava system also at work. The patient was supplied with the prescribed skin care products for use with the Brava system. Autologous fat transplantation was performed a total of 7 times over a period of 19 months. Fat transplantation volumes ranged between 30 cc and 200 cc and total transplanted fat was 957 cc. A Khouris 3 mm extraction cannula connected to a 10 cc syringe was used to harvest fat. The harvested fat was then centrifuged at 1200 rpm and the supernatant oil was removed. The prepared adipose tissue was then injected subcutaneously and into the pectoral muscle using a Coleman type 2 cannula on a 3 or 10 cc syringe. Scar tissue was subjected to rigottomy with 3 dimensional micromeshing using a 12- to 18-gauge needle. Brava therapy was resumed 8 weeks after each of the fat transfers. Furthermore, in conjunction with the aforementioned 7 autologous fat transplantations, the patient also underwent mastopexy and excision of a suture granuloma of the left breast and z-plasty of the mastectomy scar of the right breast.

**Figure 1:**
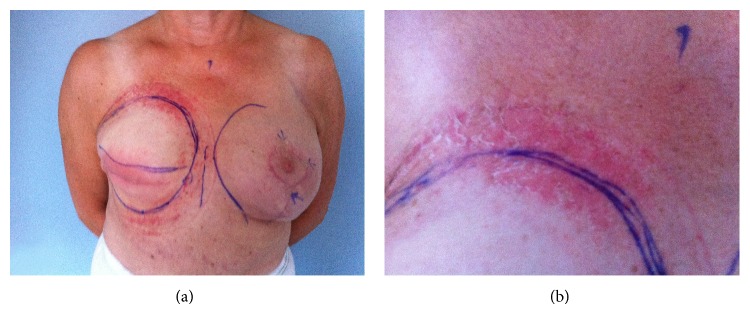
(a) Irritative skin rash located primarily to skin exposed to the Brava systems silicone gel cushioning. (b) Close-up of irritative rash.

After the second series of autologous fat transplantation, the patient developed irritative rash of the skin which had been in contact with the Brava system; the irritation of the skin persisted and increased in intensity with the continued use of Brava. After the fourth series of treatment, the evolvement of blisters resulted in sleepless nights due to pain and itching, which required prescription of sedatives and antihistamines (Figure 1). Furthermore Mildison a hydrocortisone based ointment and Comfeel Plus were applied to the skin. Due to these skin lesions, multiple additional consultations were necessary at our wound clinic. The blisters subsided only after discontinuation of Brava. A silicon allergy test was performed and found negative. Due to the pain and itching, the use of Brava was reduced with approximately 2 hours daily during the final 5 series of treatments. Aesthetic analysis was performed using preoperative and postoperative photographs. Both patient and surgeon evaluated the reconstruction of the breast as optimal with a soft and natural appearing breast after 7 series of treatment.

## 3. Discussion

Several studies have described results of Brava while only a few studies describe the effect of the Brava system on the skin [5, 7, 8]. Two studies presently published document Brava's side-effect on the skin [7, 8]. A study by Uda et al. showed that 35.7% of patients using Brava had skin lesions so severe that further treatments with Brava were discontinued [7]. Likewise a French study by Ho Quoc and Delay found that the most common symptoms from Brava were erythema and pruritus seen in 86% and 66% of patients, respectively. Blisters were present in 5% of patients of which one required a transient stop in the use of Brava [8]. Only very few cases of total or near total breast reconstruction with Brava in combination with fat transplantation are mentioned in the literature [7, 8]. In our case total breast reconstruction with Brava had rather serious side-effects on the skin of our patient resulting in the need for prescription medication, discontinuation of Brava, and multiple additional consultations. The etiology is most likely heterogenic as a result of the multiple series of fat transplantations and lack of prophylactic ointment and possibly the patient's history of a rash in conjunction with the use of adhesive scar tape made this patient more susceptible. At the Department of Plastic Surgery at Aarhus University Hospital several patients are presently undergoing treatment with the Brava system. As of this date results have been excellent in regard to volume expansion. Still we believe that Brava's dermatological side-effects warrant more focus for several reasons. First of all, subsequent to the irritative rash our patient experienced pain so significant that additional consultations were necessary. The situation thus involved both increased suffering for the patient and the cost of additional treatment incurred by the healthcare system. Secondly, because the use of Brava showed encouraging results as an alternative to more invasive flap surgery, we believe there will be an increased use of Brava and fat transplantation [2–5]. This will also be the case with regard to more extensive treatments, such as total breast reconstruction with multiple transplantations over longer periods of time. These treatments will likewise increase the demand for focus on Brava, as the skin in the recipient area will be exposed over a longer period of time. Therefore it is important that future studies describe Brava's dermatological side-effects, so as to document the extent of the problem and contribute to clinical guidance for future use of Brava and thus hopefully minimize or completely avoid these complications for future patients.
